# Ketogenic Diets in Pancreatic Cancer and Associated Cachexia: Cellular Mechanisms and Clinical Perspectives

**DOI:** 10.3390/nu13093202

**Published:** 2021-09-15

**Authors:** Natalia E. Cortez, Gerardo G. Mackenzie

**Affiliations:** Department of Nutrition, University of California, Davis, CA 95616, USA; natcortezp@ucdavis.edu

**Keywords:** ketogenic diet, pancreatic cancer, cancer cachexia, pancreatic ductal adenocarcinoma, microbiome, ketone bodies, cell metabolism

## Abstract

Pancreatic ductal adenocarcinoma (PDAC) is an aggressive and extremely therapy-resistant cancer. It is estimated that up to 80% of PDAC patients present with cachexia, a multifactorial disorder characterized by the involuntary and ongoing wasting of skeletal muscle that affects therapeutic response and survival. During the last decade, there has been an increased interest in exploring dietary interventions to complement the treatment of PDAC and associated cachexia. Ketogenic diets (KDs) have gained attention for their anti-tumor potential. Characterized by a very low carbohydrate, moderate protein, and high fat composition, this diet mimics the metabolic changes that occur in fasting. Numerous studies report that a KD reduces tumor growth and can act as an adjuvant therapy in various cancers, including pancreatic cancer. However, research on the effect and mechanisms of action of KDs on PDAC-associated cachexia is limited. In this narrative review, we summarize the evidence of the impact of KDs in PDAC treatment and cachexia mitigation. Furthermore, we discuss key cellular mechanisms that explain KDs’ potential anti-tumor and anti-cachexia effects, focusing primarily on reprogramming of cell metabolism, epigenome, and the gut microbiome. Finally, we provide a perspective on future research needed to advance KDs into clinical use.

## 1. Introduction

Pancreatic ductal adenocarcinoma (PDAC) is an aggressive and deadly disease with a five-year survival rate of ~10% [1]. Surgery, which offers the only realistic hope, is a viable option in a limited number of patients, whereas current chemotherapy and radiation therapy regimens offer minimal benefit [2]. Following diagnosis, PDAC patients experience progressive weight loss and nutritional deterioration, leading to a rapid decline in their quality of life. Approximately 80% of PDAC patients suffer from cachexia, a complex metabolic disorder characterized by loss of skeletal muscle mass (SKM). Unfortunately, there are no effective strategies to mitigate PDAC-induced cachexia. For this reason, new treatment strategies for PDAC and PDAC-associated cachexia are needed, and the exploration of dietary interventions is a critical component.

Ketogenic diets (KD) have been gaining attention for their anti-tumor, anti-inflammatory potential [3]. Characterized by a very low carbohydrate, moderate protein, and high fat composition, this diet mimics changes in the metabolism that are similar to fasting. Numerous animal studies have tested the effects of KDs on tumor growth and survival, including pancreatic cancer (PC), with the majority showing promising results [4]. Moreover, the evidence for a beneficial effect of KDs in clinical trials shows promise and merits further research [5,6]. Unfortunately, evidence on the effect and mechanisms of action of KDs on PDAC-associated cachexia is sparse. Therefore, in this review article, we discuss the potential beneficial role of KDs as a complimentary dietary therapy for PDAC treatment and associated-cachexia, and summarize the cellular mechanisms modulated by a KD.

## 2. Pancreatic Cancer: Biology and Current Treatments

Pancreatic cancer is a highly lethal disease, ranked the seventh leading cause of cancer-related mortality in the world [7]. In the United States, it is the third most common cause of cancer-related deaths, and it is estimated to become the second by 2030 [8]. PDAC is the most common and aggressive type of PC, with the lowest survival rate among all solid tumors [9]. Risk factors associated with the development and progression of PDAC are age, smoking, alcohol abuse, long-standing chronic pancreatitis, obesity, and type 2 diabetes mellitus (T2DM) [10].

The majority of PDACs are believed to arise from pancreatic intraepithelial neoplasias (PanINs), which are microscopic lesions (<5 mm) composed of flat or papillary neoplastic epithelium [11]. PanINs progression involves gradual acquisition of mutations in oncogenes and tumor suppressor genes [12]. The genes most commonly affected during PanINs progression and PDAC are KRAS (~90%), TP53 (~74%), CDKN2A (~35%), and SMAD4 (~31%) [11,13]. Besides multiple genetic/epigenetic alterations, PDAC harbors a complex and dense tumor microenvironment (TME) which acts as a physical barrier to drug perfusion, and could be responsible for drug resistance [14].

Pancreatic tumors have a remarkable resistance to conventional treatment options [15]. The main PDAC treatment is surgical resection, followed by chemotherapy, radiotherapy, and targeted therapy [14]. Although surgery is the only potentially curative treatment, tumor resection is possible in less than 20% of patients [2]. For a long time, gemcitabine (GEM) monotherapy was the primary treatment for unresectable PDAC, but the combination of 5-fluorouracil, leucovorin, irinotecan, and oxaliplatin (FOLFIRINOX), or the addition of Nab-Paclitaxel to GEM, are now the standard of care [16]. Although these combination chemotherapies are increasingly effective for PDAC, they are associated with significant toxicities and lowtumor response rates; many patients ultimately relapse and require second-line therapy [17,18,19].

## 3. PDAC-Associated Cachexia

A major contributor to PDAC mortality is cancer-associated cachexia, a complex multifactorial disorder characterized by the involuntary and ongoing wasting of SKM with or without loss of adipose tissue [20,21]. Up to 85% of PDAC patients suffer from cachexia, and around 30% of deaths are directly associated with it [22]. The diagnostic criterion for cachexia is weight loss greater than 5% within 6 months, weight loss greater than 2% in individuals with a body mass index (BMI) less than 20 kg/m^2^, or sarcopenia with more than 2% weight loss [21].

The key role of the pancreas in nutrient digestion and glucose homoeostasis, plus its interaction with other digestive organs, likely adds to the incidence and intricacy of PDAC-associated cachexia [23]. Significant preoperative weight loss is frequent even in early-stage diagnoses [24]. Tumors compete with other organs and tissues for energy fuels and biosynthetic substrates, which promotes an elevated resting energy expenditure and a negative energy balance [25]. Cachexia causes a shift in body fuel utilization where proteolysis and lipolysis are increased, but lipogenesis and SKM protein synthesis are decreased [26]. PDAC cachectic patients have an increased risk for malnutrition during chemotherapy, which in turn affects their response to treatment [27,28]. Because chemotherapeutic doses are determined by body weight or surface area, patients with a lean body mass (LBM) equivalent to underweight are more susceptible to suffering toxicities [29]. Cachexia is associated with chemotherapy toxicity, functional impairment, surgery complications, and mortality [30,31]. For these reasons, the preservation of SKM is extremely important [32]. Despite increased understanding of the mechanisms of cachexia, there is still no standard of care, no licensed drug treatment, and no evidence-based guidelines for its management [33].

**Figure 1:**
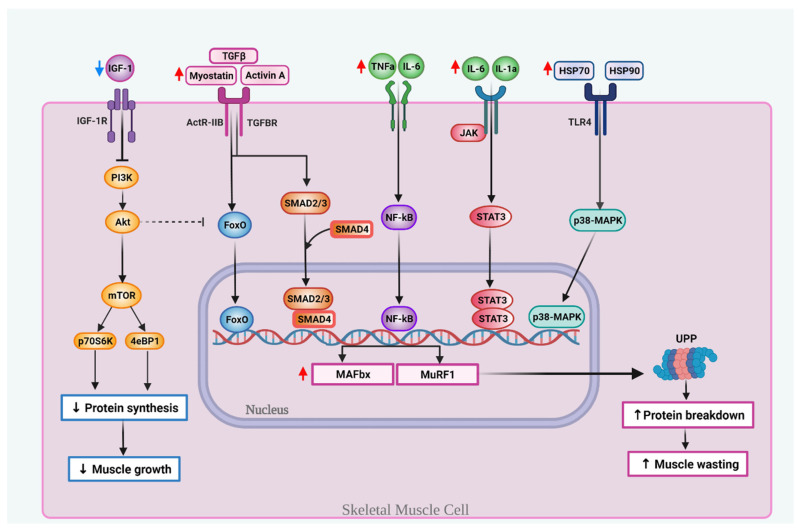
Key molecular mechanisms involved in muscle wasting during PDAC-associated cachexia. Cachexia is associated with decreased levels of insulin-like growth factor 1 (IGF-1), which inhibits protein synthesis, in part, by suppressing the PI3K-Akt-mTOR pathway. Transforming growth factor-β (TGF-β) family members, like myostatin and activin A, bind to the ActRIIB receptor complex or TGFβ receptor and activate the forkhead (FoxO) family transcription factor or Smad2/3. Smad2/3 makes a complex with Smad4. Pro-inflammatory cytokines, like tumor necrosis factor α (TNF-α), interleukin 6 (IL-6) and IL-1a can activate the nuclear factor kappa-light-chain enhancer of B cells (NF-κB) and/or the janus tyrosine kinase/signal transducer and activation of transcription (JAK/STAT). The tumor releases surface heat shock proteins Hsp70 and Hsp90 that activate the Toll-like receptor (TLR4) and upregulates p38-mitogen activated protein kinase (MAPK). The translocation to the nucleus of FoxO, SMAD2/3-4 complex, NF-κB, STAT3 or p38-MAPK induces the subsequent transcription of the muscle atrophy F-box protein (MAFBX) and muscle RING finger-containing protein 1 (MURF1), two genes that activate the ubiquitin-proteasome pathway (UPP) and induce proteolysis, which in turn increases and induces muscle-wasting.

The pathophysiology of cachexia is complex, yet likely characterized by inflammation and increased catabolism in combination with decreased muscle anabolism [33]. In addition, pancreatic tumors probably have specific mechanisms that exacerbate cachexia [24,34]. These major mechanisms implicated in the development of PDAC-associated cachexia are highlighted in Figure 1.

Multi-organ systemic inflammation occurs in both the tumor and the cachectic muscle, with signals/factors from one site affecting other sites, and with multiple deregulated signaling pathways involved [35,36]. Pro-inflammatory cytokines, such as tumor necrosis factor (TNF)-α, interleukin (IL)-1, IL-6, IL-8, interferon-γ (IFN-γ), monocyte chemoattractant protein-1 (MCP-1), and other catabolic factors (activin and myostatin) are released into circulation by the tumor [37]. These signals can, in turn, activate metabolic pathways that increase proteolysis and/or decrease protein synthesis, which culminate in SKM wasting [22]. TNF-α is a major mediator of muscle catabolism associated with poor nutritional status in PDAC patients [22,38]. IL-6, overexpressed in PDAC, correlates with cachexia, chemotherapy response, and survival [22,35,38]. Both TNF-α and IL-6 can activate the transcription factor nuclear factor kappa-light-chain-enhancer of B cells (NF-κB), known to inhibit differentiation of SKM [22,39]. Activation of the ATP-dependent ubiquitin-proteasome proteolytic pathway (UPP) causes the breakdown of myofibrillar proteins and plays a prominent role in SKM degradation in cancer cachexia. NF-κB is a major regulator of cachexia, in part, by regulating two E3 ligases of UPP: (1) muscle atrophy F box protein (MAFbx/atrogin-1) and (2) muscle RING finger-containing protein 1 (MuRF1). Of note, upregulation of MAFbx is a marker of acute muscle atrophy present in the majority of cancer cachexia cases, while MuRF1 mediates the ubiquitination of the sarcomere′s thick filament [40].

The increased levels of cytokines can also contribute to muscle-wasting and cachexia through additional mechanisms, such as the activation of janus tyrosine *kinase (JAK)*/signal transducer and activator of transcription (STAT) signaling [40]. Prolonged activation of the IL-6/JAK/STAT axis is an established mechanism in tumorigenesis and in muscle-wasting during cancer cachexia [41]. Activation of the JAK/STAT pathway correlates to a poor PDAC outcome, and increased levels of phosphorylated STAT3 are essential for muscle-wasting [42].

Transforming growth factor-β (TGF-β) family members, such as myostatin and activin A, promote muscle loss through the myostatin/activin receptor type IIB (ActRIIB). Myostatin is a key negative regulator of muscle growth, whereas activin A is involved in cell growth and differentiation [22,34]. TGF-β proteins facilitate Forkhead box O (FoxO) and SMAD2/3 activation, which in turn increase the transcription of atrophy-related genes associated with the UPP, including MAFbx/atrogin-1 and MuRF1 [40,43,44]. Interestingly, SMAD-2/3 transcription factors can also activate FoxO3 [45]. Inhibition of myostatin/activin/SMAD2/3 signaling ameliorates muscle athrophy in cancer [43]. Overall, FoxO, SMAD2/3, NF-κB, and STAT3 play a crucial role in cachexia-related muscle atrophy by triggering the activity of MuRF-1 and MAFbx [22,43,46].

The insulin/IGF-1-dependent phosphatidylinositol 3-kinase (PI3K)/Akt/mammalian target of the rapamycin (mTOR) system is a major anabolic pathway involved in muscle development and regeneration. Insulin-like growth factor binding proteins (IGFBPs) help regulate several cellular processes through the IGF-1/PI3K/AKT, NF-κB, TGF-β, and JAK-STAT pathways [34]. PDAC cachexia is characterized by lower levels of circulating anabolic factors like IGF-1 [34]. The decline in IGF-1 levels suppresses AKT activity, which is a signal that stimulates muscle growth [25]. AKT also activates mTOR complex 1 (mTORC1), consequently activating ribosomal protein S6 kinase-β1 (S6K1) and eukaryotic translation initiation factor binding protein 1 (4E-BP1), which play a dominant role in myogenesis [38]. Moreover, activated AKT inhibits FoxO3a transport, thus blocking the up-regulated expression of MAFbx and MuRF-1 [38].

Another mechanism that contributes to cachexia is related to alterations of the zinc homeostasis and the upregulation of the zinc transporters ZIP4 and ZIP14. Zinc deficiency is a common occurrence in several cancers, including PC [47]. Tumor cells exhibit dysregulated zinc uptake and efflux due to alterations in the activity of many zinc transporters [48]. For instance, the zinc transporter ZIP4 has been shown to induce tumor growth and cachexia in mice bearing orthotopic pancreatic tumors [49]. Yang et al. observed that in PC cells, ZIP4 stimulates the tumor release of surface heat shock proteins HSP70 and HSP90 via extracellular vesicles (EV), which can then activate mitogen-activated protein kinase 14 (p38 MAPK)-mediated muscle catabolism and promote cachexia. This pathway knocks down ZIP4 in PC cells mitigated by muscle-wasting and cachexia by attenuating HSP70/HSP90 release from tumor cells [49]. Another zinc transporter implicated in cachexia is ZIP14, with increased expression upregulated in muscles of mice and patients with PDAC-associated cachexia. Shakri et al. observed that ZIP14 alters zinc homeostasis, and that high levels of zinc in mature muscle fibers induce myosin heavy chain loss, therefore causing muscle breakdown. Moreover, in muscle progenitor cells, elevated zinc levels prevent muscle formation by inhibiting muscle cell differentiation. ZIP14 is up-regulated by proinflammatory conditions and can be induced by TNF-α and TGF-β [50,51]. Importantly, ZIP14 is not expressed is healthy muscle cells, suggesting that anti-ZIP14 antibodies might help prevent PDAC-associated cachexia [52].

## 4. Ketogenic Diet in Cancer

Ketogenic diets (KDs) are low-carbohydrate, moderate protein, high-fat diets characterized by the intentional restriction of dietary carbohydrate intake or nutritional ketosis, which generates ketone bodies (KBs) and induces a metabolic shift [53,54]. Given that there is no exact definition for KD macronutrient composition, its variability could serve different clinical purposes [55]. Evidence suggests that KDs produce anticonvulsive, antioxidant and anti-inflammatory effects [56]. Clinically, KDs are a treatment for epilepsy and are therapeutic in other neurological conditions such like Alzheimer, traumatic brain injury, and stroke [57].

During the last decade, there has been increased interest in evaluating the impact of KDs in cancer prevention and treatment. Based on the premise that in contrast to healthy brain tissue, brain tumors are incapable of using KBs as an energy source, KDs have been suggested as metabolic therapy for malignant gliomas and have shown to reduce neuroblastoma (NB) tumor growth and prolong survival [58,59,60]. Moreover, KDs have also shown beneficial effects in other cancer types [58,59]. A 2017 systematic review reported that KD had an inhibitory effect on tumor growth and enhanced survival time in a variety of animal cancer models [61]. For example, when gastric adenocarcinoma xenografts were fed either a KD or a standard diet *ad libitum*, survival in the KD group was significantly prolonged [62]. In mice bearing subcutaneously implanted colon tumors, a KD also had a tumor-suppressive effect [63]. In an orthotopic hepatocellular carcinoma study, tumor size, growth rate, and weight were lower in KD fed mice when compared to those fed a chow diet [64]. In pancreatic cancer xenografts, Shukla et al. showed that mice fed a KD exhibited less tumor burden along with reduced proliferation of tumor cells when compared to a chow diet [65].

In addition, when combined with existing drugs, KDs may provide a promising approach to increase the therapeutic effects of existing cancer therapies at lower levels of overall toxicity [66]. Therefore, KDs are gaining attention as potential adjuvant therapy for cancer, with numerous preclinical studies showing promising results in multiple cancer types [55,67,68]. For example, in a mouse glioma model, KD alone had no effect on median survival, but increased that of bevacizumab-treated mice [69]. In mice with two different syngeneic glioblastoma tumors grown orthotopically, efficacy against tumor growth and pro-survival was greater when the glutamine antagonist 6-diazo-5-oxo-L-norleucine was administered together with a calorically restricted KD [70]. In NB xenografts, a KD supplemented with medium-chain triglycerides (MCTs) enhanced the anti-angiogenic efficacy of cyclophosphamide [71]. In breast cancer in vitro and in vivo models, a KD in combination with melatonin showed a synergistic effect against cisplatin- and vincristine-resistance [72]. In a mouse model of anaplastic thyroid carcinoma, a KD inhibited tumor growth when in combination with the antioxidant N-acetylcysteine [73]. Furthermore, Hopkins et al. documented that feeding a KD enhanced the efficacy of PI3K inhibitors in multiple tumor models, including PC. The effect was due in part to the ability of a KD to reduce insulin feedback, which represents resistance mechanisms during PI3K inhibitor treatments [74].

KDs can also potentiate the effect of radiation. In lung cancer xenografts, KD inhibited tumor growth and extended survival by enhancing radiation response [75]. Ketocal^®^, a commercially available ketogenic formula, significantly increased survival in mice with malignant glioma when given with radiation, plus 9 out of 11 study animals were apparently cured of their implanted tumor [67]. In a PC xenograft model, KD improved radiation sensitivity and significantly increased survival [76]. Moreover, a KD seems to also improve immunotherapy. A recent study showed that a KD enhances immunotherapy in multiple types of tumor (lung, melanoma) [77]. The proposed mechanism appears to be directly related to the ability of a KD to increase 3-hydroxy-butyrate levels, which boosts the expression of CTLA-4 on CD8^+^ T cells. Additional research is needed to validate these studies in immunotherapy-resistant tumor models, such as PDAC.

Although the preclinical evidence is abundant, only a few studies have assessed the beneficial effects of KDs in humans with cancer [53]. In a study by Hagihara et al., a KD plus chemotherapy had a synergistic effect in the treatment of cancer and influenced long-term survival of advanced cancer patients [78]. Among women with ovarian or endometrial cancer, a KD might improve physical function [53]. In post-pancreatectomy cancer patients, meal compliance, energy intake rate, and meal satisfaction scores were higher in KDs compared to the general diet, with no differences in adverse events, nutritional status, and serum lipids [17]. Besides, KD appears to modulate PC-related metabolites and benefit pancreatobiliary cancer patients’ post-surgery [79]. In advanced cancer patients, a KD was shown to enhance the efficacy of chemotherapy treatment and improve quality of life [80]. The numerous clinical trials evaluating the efficacy of KD alone or in combination with chemotherapy or radiotherapy (i.e., NCT03955068, NCT01716468 NCT03535701, NCT03962647, NCT03285152) are also encouraging, including a clinical trial that is evaluating the effect of a ketogenic drink for pancreaticobiliary cancer patients (NCT03510429). A critical point to consider when analyzing the effects of KD in cancer treatment is that the macronutrient composition of the KDs varies between studies and many trials have lacked standardized protocols, so comparison among studies is difficult [58,81,82].

## 5. Ketogenic Diet in Cachexia

Cancer cachexia was believed to be resistant to nutritional interventions, yet evidence indicates that nutrition can impact it in PDAC patients [83]. Because muscle protein balance is affected by nutritional factors, some studies evaluated the impact of KDs on SKM during cancer progression [84]. The study by Shukla et al. showed that a KD can reverse PC-induced cachexia through the inhibition of systemic inflammation and muscle wasting [65]. The metabolic alterations induced by KDs, particularly the production of KBs, are associated with reduced degradation of SKM′s proteins, and decreased secretion of pro-inflammatory cytokines and metabolites involved in pathogenesis of cancer cachexia [34,80].

The favorable changes in body composition from KDs appear to be influenced by the degree of nutritional ketosis achieved [85]. KDs may exert a protective effect against muscle mass loss, potentially through maintenance rather than a net hypertrophic effect [84]. In aged mice, Wallace et al. observed that a KD resulted in preservation of SKM and increased markers of mitochondrial biogenesis, oxidative metabolism, and oxidative stress response, while decreasing protein synthesis and proteasome activity [86]. The above evidence indicates that KDs have the ability to preserve SKM and assist in mitigating cancer-associated cachexia. However, there are some conflicting reports that feeding a KD leads to muscle atrophy in mice [87]. The differences observed could be due to the composition of the KD, the length of dietary treatment, or the fact that the mice in a study by Nakao et al. lost weight, while in the study by Wallace et al., they maintained their body weight throughtout the KD intervention [86,87].

## 6. Cellular Mechanisms of KDs in PDAC and Cachexia

**Figure 2:**
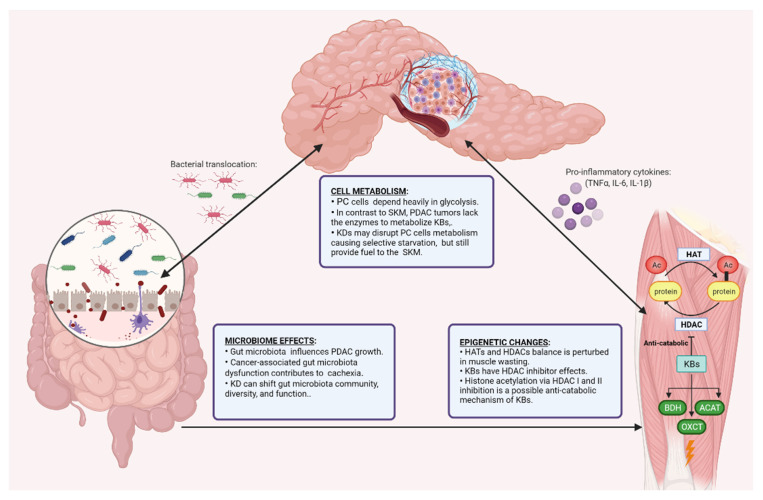
Major proposed mechanisms of the beneficial role of KDs in PDAC. The cell metabolism, epigenome, and the gut microbiome have a complex interplay in pancreatic ductal adenocarcinoma (PDAC) and associated cachexia. PDAC cells depend on glycolysis and lack enzymes that allow them to use KBs as fuel. On the other hand, skeletal muscle (SKM) has mitochondrial enzymes D-beta-hydroxybutyrate dehydrogenase (BDH1), succinyl CoA: 3-oxoacid CoA transferase 1 (OXCT1) and acetyl-CoA acetyltransferase (ACAT1), which allow KB utilization. The regulation of gene expression in cachectic SKM can be controlled by epigenetic mechanisms through acetylation and deacetylation of histones. The balance between histone acetyltransferases (HATs) and histone deacetylases (HDACs) is perturbed in muscle-wasting. A KD could modulate the gene regulation of pancreatic tumors and SKM by affecting their epigenetic state. HDAC inhibition by KBs could have anti-catabolic effects. Finally, the gut microbiome is altered in PDAC and cachexia, influencing PDAC progression and potentially cachexia. The KD can shift the microbiota profile and have a role in regulating tumor growth and cancer cachexia.

Multiple cellular mechanisms might explain the beneficial effects of a KD in PDAC and cachexia. These include anti-inflammatory, anti-angiogenesis, and cell metabolism and epigenetic effects, as well as modulation of the microbiome. Given their key role in PDAC growth and cachexia, in the sections below, we describe in more detail the effects of KDs on cell metabolism, the epigenome, and the gut microbiome as major cellular mechanisms of the beneficial role of KDs in PDAC in more detail (Figure 2).

### 6.1. Ketogenic Diet and Cell Metabolism

Metabolic reprogramming is a hallmark of cancer cells with many pathways that regulate the cell metabolism being altered. For example, the IGF1/PI3K/Akt/mTOR system is often hyperactive in cancer cells due to chronic hyperglycemia and hyperinsulinemia, as well as the mutations in genes that code for pathway proteins [88]. In PC, PI3K/AKT/mTOR and Ras/Raf/Mitogen-activated protein kinase/ERK kinase (MEK)/extracellular-signal-regulated kinase (ERK) pathways are upregulated and favor cancer cells proliferation and growth [55,89]. AMP-activated protein kinase (AMPK) regulates glycolisis and is involved in tumorigenesis and PC progression [90]. In addition, activity of lactate dehydrogenase (LDH), responsible for conversion of pyruvate to lactate, is also increased in tumor cells [91]. LDH is a target gene of c-Myc, an important regulator of glucose metabolism, cell growth, and proliferation, and is frequently amplified in PDAC [92,93,94]. In PDAC, high serum LDH levels are a poor prognostic indicator [92]. Pyruvate kinase (PK) regulates the final rate-limiting step of glycolysis. PK isoform M2 has a critical role in reprogramming cancer metabolism, is upregulated in various tumors, and contributes to tumor growth and angiogenesis by regulating hypoxia-inducible factor 1 (HIF-1α) [95]. Furthermore, genes involved in glycolysis and glycolytic transport to the mitochondria are dysregulated in tumor cells and cause mitochondrial alterations [67].

Several mechanisms support the rationale of using a KD to modulate cancer metabolism [96,97]. KDs mimic many of the metabolic and anti-inflammatory properties of calorie restriction (CR), including reduced blood glucose, insulin, and IGF-1, as well as the oxidation of fatty acids and generation of ketones [98]. Decreased blood insulin/IGF-1 levels by KD lead to an inhibition of the PI3K/Akt/mTOR and Ras/Raf/MEK/ERK cascades [65,98]. Hyperglycemia inhibits AMPK, which in turn activates mTOR, but ketosis activates AMPK and inhibits mTOR [99,100,101]. Importantly, KBs can be used by healthy cells as energy sources, but many tumors are largely unable to metabolize them [102]. Therefore, KD tumor-suppressive effects are mainly based on the Warburg effect [55]. Since cancer cells depend heavily on glycolysis, by reducing glucose, insulin, IGF-1 levels, plus lactate production, KDs potentially induce selective starvation in cancer cells [92,103]. Shukla et al. showed that treatment with KBs reduced expression of GLUT1, LDH, and c-Myc in PC cells, which might contribute to the inhibiting effects of KBs in PDAC growth [65].

In addition, fatty acid oxidation occurs primarily in the mitochondria and is dependent upon efficient and well-integrated mitochondrial electron transport chain activity [76]. Ketosis decreases ROS production in healthy tissues [96,104]. Therefore, KDs may disrupt cancer cell metabolism and limit the energy source of the tumors, while still providing fuel for the host [105]. Results from the studies by Chang et al. showed that malignant gliomas have differential expression of ketolytic and glycolytic enzymes, and suggest that expression profiles could potentially be useful as biomarkers for KD therapy response [106]. Consistently, Zhang et al. demonstrated that xenograft tumors expressing low levels of BDH1 and OXCT1 are more responsive to KD therapy. In their studies, KD inhibited growth of Panc-1 and significantly prolonged the mean survival of mice with Panc-1 xenograft tumors. However, in mice with HeLa xenografts KD increased tumor growth and significantly lowered survival, suggesting that pancreatic cancer cells are more responsive to KD therapy, likely due to their lesser ability to metabolize KBs [107]. Besides ketolytic enzyme expression, the timing of KD administration may play a role in the effects of KDs against tumor growth. A meta-analysis by Klement et al., described that mice that were in ketosis at the time of tumor cell injection seemed more protected than those receiving KD after tumor transplantation [108].

**Figure 3:**
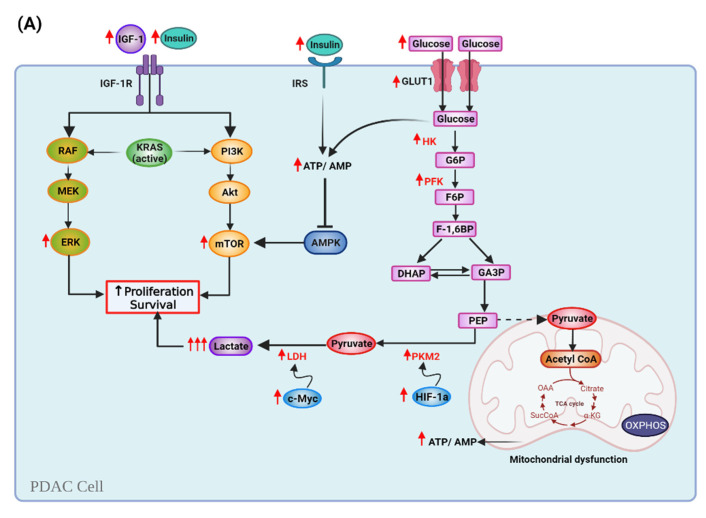

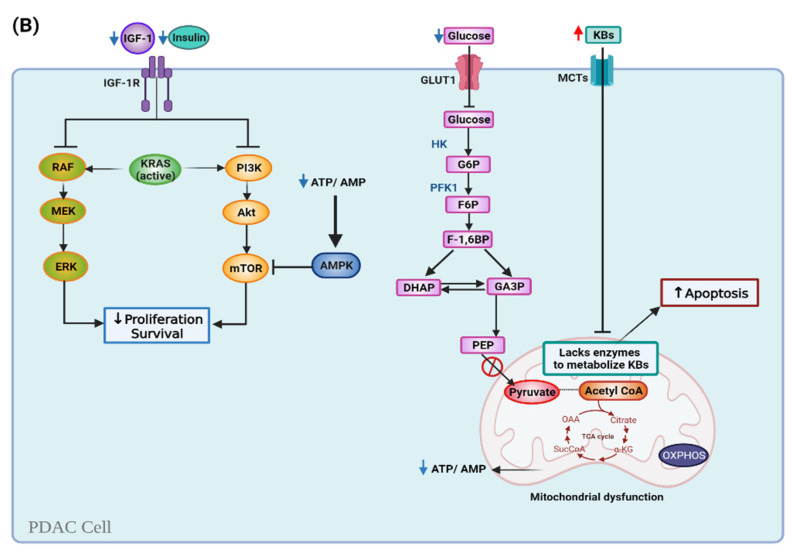
Pancreatic cancer cell metabolism during a normal carbohydrate-rich diet versus a ketogenic diet. (**A**) Cancer cells undergo various metabolic modifications to satisfy their energy needs. High levels of insulin and insulin-like growth factor-I levels (IGF) induce upregulation of insulin/IGF-1-dependent phosphatidylinositol 3-kinase (PI3K)/Akt/mammalian target of the rapamycin (mTOR) system and the Ras/Raf/Mitogen-activated protein kinase/ERK kinase (MEK)/extracellular-signal-regulated kinase (ERK) cascade. KRAS mutation affects glucose dependency. The expression of glucose transporter 1 (GLUT1) is stimulated, increasing glucose uptake and glycolysis. Pyruvate kinase isoform M2 (PKM2) and lactate dehydrogenase (LDH) are over-expressed, so lactate levels increase. Hyperglycemia inhibits AMP-activated protein kinase (AMPK), which in term activates mTOR (**B**) KDs reduce circulating glucose, which halts glycolysis. Decreased blood glucose, insulin, and IGF-I levels inhibit the PI3K/Akt/mTOR pathway, and lactate production, therefore inducing selective starvation in cancer cells, and targeting proliferation and survival. Ketosis activates AMPK, which inhibits mTOR. Mitochondrial dysfunction and lack of the mitochondrial enzymes that metabolize ketone bodies (KBs) cause the mitochondria to decrease ATP production. Note: Upward red arrows mean upregulation; downward blue arrows mean downregulation.

A summary of the Warburg effect and metabolic reprogramming in PDAC cells in the presence or absence of glucose is shown in Figure 3.

Although malignant cells lack key mitochondrial enzymes required to metabolize KBs, muscle cells maintain this ability [109,110]. CR facilitates mitochondrial fat oxidation in SKM through upregulated AMPK signaling, working in opposition to IGF-1-mediated activation of mTOR [111]. KDs may contribute to SKM maintenance or growth through upregulation of mTOR signaling [85,112]. Huang et al. showed that a normal-protein KD activated mTOR-related proteins that drive protein synthesis over autophagy [85]. In addition, Roberts et al. showed that, in aged mice, a KD preserved muscle mass and motor function by increasing protein acetylation levels and modulating mTORC1 signaling in a tissue-dependent manner [113]. In addition, a KD can modulate amino acid metabolism. Douris et al. documented that consuming a KD for 80 weeks decreases amino acid catabolism in mice, with animals maintaining an improved glucose homeostasis, and showing a reduction in the hepatic expression of genes responsible for amino acid catabolism including branch-chain amino acids catabolism [114].

The previously discussed metabolic alterations in cancer cells contribute to the secretion of cytokines and metabolites involved in cancer-induced cachexia, and the catabolism of the SKM can then provide metabolites and energy sources for tumor growth. Conversely, the metabolic alterations induced by the KD lead to decreased secretion of pro-inflammatory cytokines and metabolites associated with cachexia [34]. In mice with PC xenografts, significantly lower glucose concentration and tumor weight, plus significantly higher βHB, muscle weight, and carcass weight was observed in a group fed a KD when compared to a group fed standard chow. Therefore, KD may be associated with diminishing tumor growth and inhibiting cancer-induced cachexia [65]. The anti-cachectic effects of the KD are not surprising considering that during prolonged fasting or starvation there is a metabolic shift to fat metabolism and ketosis in order to spare protein [80].

As KBs reduce inflammation and oxidative stress, they also may have anti-catabolic effects during inflammation-related muscle atrophy [115]. Previous studies have suggested that βHB has potent anti-catabolic actions in muscle during acute inflammation [116]. Nakamura et al. demonstrated that the elevation of plasma IL-6 concentration was inhibited when given Ketonformula, which, together with increased βHB levels, suppressed systemic inflammatory responses and colon tumor progression, in turn improving body and muscle weight [68]. Additionally, KDs may strengthen bioenergetic signaling that upregulates mitochondrial fat oxidation and endogenous antioxidant defense and downregulates inflammation [111]. In sedentary rats, a KD improved oxidative capacity and aerobic energy metabolism in muscle, without compromising muscle performance [117].

### 6.2. Ketogenic Diet and the Epigenome

Epigenetic alterations are emerging mechanisms of cancer progression, including PDAC [118,119]. The epigenetic modifications of the genome involve changes to chromatin structure, DNA methylation, and histone modification, amongst the main ones. Interestingly, stage specific DNA methylation, and histone modifications have been detected in early tumorigenesis, PDAC progression and metastasis [119,120]. KD could inhibit tumor growth and/or improve the effectiveness of cancer therapies through epigenetic modifications, such as DNA methylation, that affect the regulation of genes involved in tumor survival and proliferation [55,66].

Besides epigenetic changes at the tumor sites, the regulation of gene expression in cachectic SKM can also be controlled by epigenetic mechanisms through acetylation and deacetylation of histones, which are then modified in a post-translational manner through histone acetyltransferases (HATs) and histone deacetylases (HDACs) [121]. HDACs are enzymes that remove acetyl groups and condense chromatin. Sirtuins (SIRTs) are also capable of histone deacetylation [122]. Histone acetylation plays numerous roles in muscle control and different classes of histone deactylase have different effects on SKM [84]. Histone acetylation via Classes I and II HDAC inhibition is a possible anti-catabolic mechanism of ketones [115]. Interestingly, the balance between HATs and HDACs is perturbed in muscle wasting [123]. Hyperacetylation of transcription factors and nuclear cofactors regulating gene transcription in muscle-wasting may influence muscle mass. In addition, hyperacetylation may render proteins susceptible to degradation by different mechanisms, including intrinsic ubiquitin ligase activity exerted by HATs and by dissociation of proteins from cellular chaperones. In recent studies, inhibition of p300/HAT expression and activity and stimulation of SIRT1-dependent HDAC activity reduced glucocorticoid-induced catabolic response in SKM, providing further evidence that hyperacetylation plays a key role in muscle-wasting. Although epigenetic markers are inherited, there is evidence that some can be modified by environmental variables, including diet [122,124]. HDAC inhibitors are capable of reducing cancer cell proliferation and enhancing programmed cell death, and βHB and ACA have demonstrated HDAC inhibitor effects [66].

At the molecular level, βHB and ACA affect epigenetic marks by inhibiting HDAC1, and this modulates protein expression at the post-translational level, affecting DNA methylation and acetylating histone and non-histone proteins [125,126,127]. Of note, evidence also suggests that βHB can have a direct epigenetic effect via a novel histone modification known as β-hydroxybutyrlation of H3K9, which results in improved gene regulation [128]. KBs affect epigenetic marks by inhibiting HDAC1, modifying proteins at the post-translational level by butyrylation, affecting DNA methylation and acetylating histone and non-histone proteins. Epigenetic changes stimulated by KBs potentially modulate the expression of proteins involved in carcinogenic pathways [55].

The epigenetic effect of a KD might be due to the production of βHB. Shimazu et al. have shown that in the brain, βHB is more than an energy molecule; it plays important roles in cell signaling. The signaling functions of βHB are linked to the epigenetic regulation, as it is an endogenous class 1 HDAC inhibitor [126]. Another postulated epigenetic mechanism could be associated with the ability of a KD to increase adenosine levels. A study in epileptic rats fed a KD observed reduced gene expression due to ameliorated DNA methylation and increased adenosine levels, which blocks DNA methylation [129]. Roberts et al. showed that a KD extends median survival in mice, in part, by increasing protein (and histone) acetylation [113]. In addition, KD has been linked to increased global histone acetylation, with a specific increase in the expression of protective genes, such as Foxo3a, which plays an important role in cachexia. Finally, the epigenetic regulations by KDs have been postulated to affect patient treatment response [118,130].

Furthermore, recent studies have indicated the importance of the TME on tumor growth, as well as in PDAC treatment response [131]. KD could also modulate the activity of the pancreatic TME by affecting their epigenetic state. Wallace et al. reported increased markers of neuromuscular junction turnover, mitochondrial biogenesis, oxidative metabolism, and oxidative stress response, and decreased ER stress, protein synthesis, and proteasome activity as mechanisms through which a KD results in the preservation of SKM and function in mice [86].

### 6.3. Ketogenic Diet and the Gut Microbiome

The role of the microbiota in mediating the anti-tumor effects of KDs still needs to be fully investigated. Such a role appears possible given findings that indicate an important contribution of the gut microbiome to PDAC growth and treatment [132]. Emerging evidence also indicates that the gut microbiota is an important mediator of PDAC progression [133]. Individuals with PDAC appear to have microbial alterations with increased *Bacteriodetes,* but decreased *Firmicutes* and *Proteobacteria* compared to healthy controls [134]. Furthermore, ablation of the gut microbiota with wide-spectrum antibiotics reduced the PC burden in a murine xenograft model [135]. Several strategies are being explored to modulate the gut microbiome in PDAC patients in order to shift the TME from an immunosuppressive to an immune-active state [132]. Riquelme et al. demonstrated that human fecal microbial transplantation positively affects PDAC tumors by modulating the gut microbiota and the immune system [136].

Several studies have explored the effect of a KD on the gut microbiome. Interestingly, a KD reversed the dysbiosis associated with certain neurological disorders, including autism, multiple sclerosis, and refractory epilepsy [137,138,139]. Moreover, individuals consuming a KD for 8 weeks significantly shifted their gut microbiota community, diversity, and function in a manner distinctive to high-fat diets. Moreover, KD decreased the gut and adipose tissue levels of pro-inflammatory Th17 cells [140]. In infants with refractory epilepsy, an increased proportion of circulating Th17 cells was partially reversed following KD consumption [141]. These data provide a premise for future investigations into the potential role of immune responses as a mechanism underlying the efficacy of a KD. Along these lines, the beneficial effects of a KD alone or in combination with chemotherapy/immunotherapy in PDAC and its impact on the microbiota is yet to be explored.

Besides its role in regulating tumor growth, the gut microbiota may play an important function in the context of cancer cachexia. Cancer-associated gut microbiota dysfunction can alter mitochondrial energy metabolism in SKM, contributing to the negative energy balance in cachectic patients. The amino acid bioavailability and metabolites generated by the gut microbiota can influence energy expenditure in the muscle cells [40]. In addition, the gut microbiota may influence the efficacy of chemotherapeutic agents utilized in PDAC. For example, some bacteria species have the ability to metabolize GEM and decrease its activity [142]. Hence, modulation of the gut microbiota could potentially sensitize the tumor to chemotherapy, and has potential as a therapeutic target in the modulation of disease progression [133]. In cancer cachexia, gut barrier function and microbiota composition appeared to be altered independently of chemotherapy [143]. However, the mediators of PDAC-induced cachexia, PDAC treatment, and their interplay with microbiota still remain elusive, and additional research is warranted to evaluate whether select microbiome changes induced by a KD could be beneficial in PDAC.

Another important aspect to consider in future studies is the determination of the microbiome profile in each PDAC patient. This information will guide the implementation of specific diets, such as KDs, based on the species comprising their microbiome. One caveat is the need to monitor the gut microbiome regularly after the start of the diet to assess whether it was successful in modulating the gut microbiome profile. If the dietary intervention does not exert a strong and continuous effect, fecal microbiota transplants might be needed to fully change the microbiome at the start of the intervention, which could then be further sustained and enhanced through a KD. Nevertheless, additional research is needed in this area to determine the impact of KDs on the gut microbiome.

## 7. Clinical Perspectives and Future Directions

Although KDs are an established therapy for epilepsy in humans, the evidence for a clinical beneficial effect of KDs for cancer patients is less consistent, but nonetheless promising [110,144]. KDs seem to be most beneficial when used as an adjuvant therapy with other treatment strategies. Even though many studies included only a small number of participating patients, they have provided promising indications that a KD is safe, feasible, and improves outcomes in patients with several types of advanced cancer [5,6].

Regarding its effect mitigating cancer-cachexia, a majority of the studies indicate that a KD preserves muscle mass, suggesting that it might prove to be instrumental in limiting cachexia development. Unfortunately, the current evidence in clinically-relevant PDAC models and/or human trials is limited, and further work is required to elucidate applicability of a KD in PDAC-cachectic patients. Given that the evidence suggests that the effect of the diet increases with time, investigations at an earlier stage in disease progression are warranted to evaluate long-term effect of KDs in mitigating PDAC cachexia.

Even though we have made significant progress in the understanding of the cellular mechanisms of a KD in cancer and cachexia, important questions remain unanswered. There is no standardized KD, thus, variability in compositions (fat, protein and carbohydrates levels) may lead to different effects. Therefore, a standardized treatment protocol that includes the length and regimen for a KD remains to be established [110].

A major limitation of any dietary intervention is its compliance over a long period of time. Indeed, strict diets are difficult to maintain throughout cancer treatment. Thus, it is important to consider whether an intermittent KD schedules might have a beneficial effect similar to those observed using a strict KD. This possibility has been recently investigated and the authors observed that intermittent administration of KBs induced T-cell dependent tumor growth inhibition [77]. Another possibility that requires further evaluation is whether dietary supplements that increase blood ketone levels, such as a ketone esters (KE), provide the physiological benefits of ketosis without extreme dietary restrictions. For example, in animals and humans, feeding a KE-diet [30% kcal from (*R*)-3-hydroxybutyl (*R*)-3-hydroxybutyrate] have shown to increase circulating βHB concentrations and lowered plasma cholesterol, triglyceride, and glucose levels, compared with pair-fed, isocaloric diets in which the KE was replaced by fat or carbohydrate [145]. Importantly, the authors showed that feeding a KE-diet enhanced motor and physical performance [145]. Of note, KEs are proven to be safe in rats [146], and humans [147]. Thus, although studies in cancer models are lacking, this evidence supports the possibility that a KE-die might be beneficial for cancer-associated cachexia.

An additional concern of the applicability of a KD in PDAC patients is that anorexia, in addition to the side effects of chemotherapy (nausea, vomit, diarrhea, dysgeusia) contribute to a high risk of malnutrition in PC patients [48,148,149]. Patients following a KD have reported constipation, diarrhea and fatigue, and other less common effects, such as increased level of low-density lipoprotein cholesterol and shakiness, but no significant adverse effects [150]. Moreover, the anatomical location of the pancreatic tumor (i.e., head of the pancreas) might affect the digestion of lipids due to an obstruction of the secretion of digestive enzymes into the duodenum. This may represent a major problem for proper lipid digestion and absorption, in particular when consuming high amounts of fat. Indeed, pancreatic exocrine insufficiency (PEI) often occurs with pancreatic tumors [148], contributes to cachexia and correlates with poor survival in advanced PDAC patients [151]. Due to the high prevalence of PEI in PDAC, patients usually receive pancreatic enzyme replacement therapy (PERT), the standard therapy for PEI, which is associated with weight maintenance and increased survival in PC patients [148,152,153]. However, PERT prescription needs to be individualized and residual pancreatic function, clinical data, nutritional parameters, and dietary fat intake considered [153,154]. Thus, these commons clinical practices (such as PERT) should be considered when implementing KD interventions in PDAC patients.

As the field of precision nutrition/medicine gains traction, it is becoming more evident that a “systems biology” approach is necessary. Given the heterogeneity of PDAC tumors and their complex TME, it appears critical to genotype each tumor to determine its metabolic profile. As KDs are gaining in popularity, genetic variants for the prediction of a KD response must be considered [155].

In summary, the evidence to date strongly suggests that KD strategies are beneficial in PDAC and PDAC-associated cachexia, particularly when used adjuvant to treatment. We believe that in order to advance in the implementation of KD strategies, research should focus on understanding the long-term effects of KDs in humans bearing PDAC. Aside from physiological factors like age and gender, future studies should consider additional factors, such as lifestyle, dietary intake, body composition, gut microbiome composition, genotype, epigenetics, and the interplay of all factors, in order to maximize a patient′s likelihood of successful response to KDs in combination with the standard of care.

## Data Availability

Not applicable.
